# Ophthalmology and Otology

**Published:** 1894-01

**Authors:** Alvin A. Hubbell

**Affiliations:** Professor of Ophthalmology and Otology, Niagara University


### OPHTHALMOLOGY AND OTOLOGY.

Conducted by ALVIN A. HUBBELL, M. D.,
Professor of Ophthalmology and Otology, Niagara University.

### PATHOLOGY OF HYPOPYON KERATITIS.

Marple (*Archives of Ophthalmology*, October, 1893,) reports a case of hypopyon keratitis with enucleation of the ball and subsequent microscopical examination. He attempts to account for the origin and formation of pus in the anterior chamber—a problem that has not been satisfactorily solved. His conclusions are "that the pus in the hypopyon comes, in the majority of cases, from the uveal tract, the iris and the vessels adjacent to Fontana's spaces, while that in the cornea is derived either from the deep ciliary vessels or the anterior border-loop vessels. So much is positive. As to the source of the pus-cells, which appear in the exudate on Descemet's membrane very early in most cases, we are still uncertain. The pus in the cornea may be derived from the conjunctival sac, and may originate from the cornea itself. At least it has not been proven that it does not."

---

CROUPOUS IRITIS.

DR. ADOLF ALT, of St. Louis, (*Journal of Ophthalmology*, November, 1893,) describes a form of iritis which he terms "croupous." It is that form which Knapp has designated as "spongy iritis," and is scarcely mentioned in the text-books. The literature of the subject began with Schmidt, who reported two cases in 1871. Since then it has been noted by Gunning, Gruening, Kipp, Knapp, Alt, and S. M. Burnett. This is an inflammatory disease of the iris, ushered in by severe pain in and about the eye, edema of the lids and conjunctiva, and circumcorneal injection. This inflammation leads to a peculiar exudation into the anterior chamber, but which is in no way distinguishable from croupous exudation elsewhere. It forms rapidly, and is first seen as a grayish, grayish-yellow, or grayish-green semi-transparent substance (showing sometimes stripes and dots), which, when the patient is seen, usually fills the anterior chamber to its full extent. After a period varying from a day to a week, and even much more, during which time hemorrhages into the anterior chamber not infrequently take place, the exudation becomes transparent in its periphery, and is liquified and gradually absorbed. This change is visible usually at first in the upper part of the anterior chamber, where a small strip of iris-tissue becomes uncovered, the exudation sinking by gravitation. It then presents a sharp, well-defined, sometimes perfectly round, sometimes jagged edge upward, and has at this stage an appearance much like that of a cataractous lens, dislocated into the anterior chamber. This resemblance is the more striking, as the anterior chamber is usually very deep. Gradually the absorbing and melting process goes on, till later only a small piece is seen lying at the bottom of the anterior chamber, and finally after ten to twenty-five days it entirely disappears. The eye then quickly recovers, only one or more posterior synechia remaining to mark the disease. In uncomplicated cases not even a synechia may remain.

It seems highly probable that we have to deal with a special form of infection which is worthy of further study. In treatment the same measures may be used as in plastic iritis.

ERRORS OF REFRACTION AND THEIR CORRECTION IN EPILEPTICS.

Mr. Work Dodd read a paper on this subject before the Ophthalmological Society of the United Kingdom, (*Medical Week*, October 27, 1893,) which was the outcome of a study of 100 cases. The refractions were worked out under mydriatics with every care, under the following conditions: (1) The total refraction under a mydriatic was taken; (2) hypermetropia of 0.75 D was reckoned as emmetropia; (3) astigmatism of 0.25 D was not included.

The following are the results of his researches: Emmetropia, 7; simple hypermetropia, 42; simple myopia, 6; total astigmatism, 42, of which there were simple hypermetropic astigmatism, 3; compound hypermetropic astigmatism, 24; compound myopic astigmatism, 2; mixed astigmatism, 6; marked anisometropic astigmatism, 7; other cases of marked anisometropia, 3. If we compare this table with a classification of percentages of refraction, obtained from fifty cases of apparently normal eyes, which were worked out under mydriatics in the same manner, we find emmetropia, 6; simple hypermetropia, 70; simple myopia, 2; total astigmatism, 16. It will be seen that the most marked difference is in the amounts of the simple hypermetropia and of simple astigmatism, there being twenty-eight cases per cent. less in the epileptic than in the apparently normal class. Of astigmatism of all kinds there are twenty-six cases per cent. more in the epileptic division than in the normal one, chiefly made up by the large amount of compound hypermetropic astigmatism existing in epileptics.

Of the 100 consecutive cases of epilepsy, seventy-five were ordered to wear glasses; of these there were twenty-three who either did not wear them, or failed to report themselves later and could not be found. Of the remaining fifty-two cases there were: (1) Thirteen who had no fits since using the glasses, during periods varying from four months to one year; (2) three patients whose condition had not apparently altered; and (3) thirty-six patients whose condition had improved since wearing glasses; in the majority of these the improvement had been marked. In all the cases the ordinary treatment was continued for some time. Several cases in which patients who had ceased to have fits since wearing the glasses, had suffered from them again through some other form of irritation, but in no case had the fits been as severe as before.

Mr. Dodd thinks that we may deduce from the foregoing facts that, given a certain condition of instability of the nervous system, errors of refraction may excite epilepsy, and their correction, with other treatment, may relieve and even cure it.

---

### THE TREATMENT OF ULCERS AND ABSCESSES OF THE CORNEA BY CURETTING AND IRRIGATION.

DeWecker (*Annales d'Oculistique*, July, 1893,) has practised curetting and antiseptic irrigation of corneal ulcers and abscesses for some time with surprising results, of which the following are the most marked :

1. The suppression, sometimes instantaneous, of pain and photophobia.

2. The clearing up of the surrounding parts, followed by a cure infinitely more rapid than is obtained by the employment of various antiseptics and particularly the actual cautery.

3. Reparation by a more transparent tissue is better obtained by this method than by any other mode of treatment.

He operates by using sharp curettes of small dimensions, and of various forms, of which one differs but little from that of Critchett, except that it is one-third as wide and its edge is sharp. He endeavors as much as possible to remove from the bottom and edges of the ulcer all the adherent whitish parts in such a way that, under the jet of the irrigator (charged with a four per cent. solution of boric acid), the parts of the cornea not attacked by the curette appear feebly opaline, but uniformly transparent.

He does not hesitate to affirm that the curetting (*raclage*), joined with irrigation, should be used by all clinicians until the progress of our therapeutics furnishes something better.

---

### FORMIC ALDEHYDE AS AN OCULAR ANTISEPTIC.

Valude, of Paris, (*Annales d'Oculistique*, July, 1893,) has been led to study the antiseptic properties of formic aldehyde, and believes he has found in this a powerful antiseptic, and, being but little irritating, one that is particularly suited to the eye. He recommends it in cases to be operated upon, in post-operative infection, in purulent affections of the eye, and in the sterilization of collyria, as it does not precipitate the alkaloids (atropine, eserine, cocaine,) as does bichloride of mercury. He uses it in the eye in solutions of 1 to 100 to 1 to 500, and to preserve collyria 1 to 2000.

Finally, as it does not attack metals—steel, silver, or aluminum—it may be used for washing and disinfecting instruments, in solution, for example, of 1 to 500.

---

### REMOVAL OF THE STAPES IN CHRONIC NON-SUPPURATIVE DISEASE OF THE MIDDLE EAR.

Dr. Clarence J. Blake, of Boston, (*Archives of Otology*, 1893,) has recorded his experience in the removal of the stapes for chronic, non-suppurative catarrh of the middle ear. His conclusions are so important that they are here presented in full:

"In reviewing the cases reported, . . . it is very evident, so far as conclusions can be drawn from a small number of cases, that the operation of the removal of the stapes does not answer the purpose which might be hoped from it in cases of chronic non-suppurative disease of the middle ear. This conclusion is one in which the clinical and operative observations are entirely in accord with the pathology of this class of cases as set forth by numerous observers, and lastly and most clearly by Politzer. For all this class of cases, therefore, I should, as the expression of a personal opinion and as the result of experience, advise an exploratory tympanotomy with local and without general anesthesia, as a preliminary to, or as the first part of, an operation having in view any form of interference with the middle ear, from simple mobilization of the ossicular chain to the removal of the stapes.

"The exploratory tympanotomy, especially where the incision is made, as it should be, close to the periphery of the membrana tympani and of sufficient extent, affords an opportunity for a better determination of the condition of the middle ear in chronic non-suppurative disease than can be obtained in any other way, and after the exploratory incision, if it seems advisable not to operate more extensively, the opening in the membrana tympani can be closed by a simple paper dressing, with the prospect of speedy healing. If, however, the exploratory operation and coincident tests show that it is advisable to perform an operation in the middle ear, whether synectomy, tenotomy, incudectomy, incudo-stapedectomy, or stapedectomy, the opening suffices for the purpose.

"In the great majority of the cases of stapes fixation, consequent upon chronic non-suppurative disease of the middle ear, the operation . . . was ineffectual, so far as the removel of the stapes was concerned, the fixation of the base-plate at least being such as to result in fracture of the crura instead of the removal of the ossicle entire. In all the cases of non-suppurative disease in which the stapes was extracted entire, the hearing was definitely and practically improved in one only; and of the two other cases in which definite improvement in hearing resulted from the operation, there was one in which the mobilization of the base-plate, incident to the fracture of the crura, gave an improvement for high tones, and for the voice in ordinary conversation only to the extent of about twenty per cent.

"When we take into consideration the secondary changes which may have occurred in the internal ear in the course of a non-suppurative disease of the tympanum, and the injury to the delicate structures in the labyrinth, which might result from the force exerted in the extraction of the stapes, coupled with the inadequate results as set forth in the experience tabulated, it may be justly said that stapedectomy does not afford a promising outlook for this class of cases."

### AURAL REFLEX OF UNUSUAL CHARACTER, DUE TO IMPACTED WAX.

THEOBOLD (*New York Medical Record*, July 29, 1893,) reports the case of a female, aged 42, who had suffered for six months with an annoying cough and with spells of inability to swallow food. These symptoms were increased on manipulation of the right ear. Examination showed a piece of wax which had been forced against the drum-membrane by attempts at removal. It was removed by syringing, and the difficulty in swallowing and other symptoms disappeared.

---

THE hostess who sends a pitcher of ice water to her guest's room should use as large a lump of ice as will fit the pitcher, and not too much water; then set the pitcher in the center of a large newspaper, gathering the ends up at the top and place a strong rubber band around them, to exclude the air. Treated in this way, the water will remain real ice water all night, and a lump of ice as big as one's fist will not be entirely melted by the next morning. —*Buffalo Commercial.*

